# Telemedicine for under-resourced patients with systemic lupus erythematosus: a qualitative study exploring the views and experiences of patients and their healthcare team

**DOI:** 10.3389/frhs.2025.1503881

**Published:** 2025-05-22

**Authors:** Savannah Bowman, Sandeep K. Agarwal, Keshia C. Ferguson, Maryjo J. Maliekel, Maria A. Lopez-Olivo, Maria E. Suarez-Almazor, Maria I. Danila, Jinoos Yazdany, Sebastian Bruera

**Affiliations:** ^1^ Section of Immunology, Allergy, and Rheumatology, Baylor College of Medicine, Houston, TX, United States; ^2^ Department of Health Services Research, University of Texas MD Anderson Cancer Center, Houston, TX, United States; ^3^ Division of Clinical Immunology and Rheumatology, University of Alabama at Birmingham, Birmingham, AL, United States; ^4^ Birmingham Atlanta Geriatrics Research Education and Clinical Center (GRECC), Birmingham VA Medical Center, Birmingham, AL, United States; ^5^ Division of Rheumatology, University of California, San Francisco, CA, United States

**Keywords:** telemedicine, systemic lupus—erythematosus, socioeconomic disparity, qualitative, thematic analyses

## Abstract

**Objectives:**

There is scarce knowledge on the benefits, limitations, and acceptance of telemedicine in patients with systemic lupus erythematosus (SLE), particularly in those from under-resourced groups. We aimed to assess the experiences and views on telemedicine of people with SLE, clinicians, and nursing staff from a safety net healthcare system in Harris County, Texas, defined as a hospital network that primarily serves low-income, uninsured, and vulnerable populations.

**Methods:**

We conducted semi-structured 1:1 in-person interviews of patients with SLE and their clinical team members, in Harris County, Texas. Using Levesque's conceptual framework for healthcare access, semi-structured interviews and content analysis were used to explore benefits and limitations of telemedicine in under-resourced patients with SLE. Interview content was coded using inductive and deductive approaches. Data collected proceeded until thematic saturation was reached. Themes and subthemes were identified and visualized.

**Results:**

Fourteen interviews were conducted. The participants included six patients with SLE, three rheumatologists, two nurses, two medical assistants, and one rheumatology fellow. All participants had previously participated in telemedicine visits. One hundred and fifty-one codes were identified. Five key themes emerged from the analysis, including: (1) Access and Convenience, (2) Technological and Linguistic Barriers, (3) Economic Considerations, (4) Quality of Care and Disease Outcomes, and (5) Implementation of Telemedicine. Analysis showed that telemedicine could improve access to care and adherence to clinic visits by reducing the barriers associated with socioeconomic factors. On the other hand, barriers to telemedicine included digital literacy, concern about negative impact on physician-patient relationship, and language discordance.

**Conclusion:**

There is an opportunity to improve access to care in patients with SLE, particularly from under-resourced backgrounds, by leveraging the benefits of telemedicine with respect to access to care, while addressing the barriers to successful implementation.

## Introduction

Systemic lupus erythematosus (SLE) is a chronic autoimmune disease associated with high morbidity and damage accrual. Clinicians caring for patients with SLE frequently utilize clinical and laboratory evaluations to aid in the assessment of disease activity and monitor for treatment-related adverse effects. The 2019 European League Against Rheumatism (EULAR) guidelines recommend laboratory evaluation at each clinic visit every three months (1). These frequent assessments may be burdensome to patients, particularly those who have difficulties with transportation, childcare or family care needs, and work schedules. Clinic no show rates in patients with SLE are high, especially in those with low socioeconomic status, a group in which no show rates has been reported to be as high as 58.8% over one year (2). Low socioeconomic status and renal involvement have been associated with non-adherence to clinic visits in patients with SLE (3). As non-adherence to clinic follow-up visits and treatment can result in higher rates of renal failure, emergency room visits, and hospitalizations in patients with SLE, there is a need to identify models of care to improve clinic visit adherence (4, 5).

Telemedicine may provide the opportunity to improve access to care and decrease no show rates in patients with SLE. The coronavirus 19 (COVID 19) pandemic resulted in the widespread use of telemedicine as a modality for clinic visits including rheumatology clinic visits. A randomized controlled trial from Hong Kong during the COVID-19 pandemic compared telemedicine follow up to standard in-person follow up in patients with lupus nephritis (6). This study showed that telemedicine resulted in better patient satisfaction and similar short term disease control. However, telemedicine was associated with more hospitalizations, especially in patients with higher physician global assessment scores. A prospective cohort study from Singapore, comparing telemedicine visits to physical visits found no difference between SLE disease activity and flares between the two groups (7). In addition, adjustments in corticosteroid dosages, were similar between the telemedicine and in-person visit groups. Additional studies have shown that both patients with rheumatic conditions and those without were overall satisfied with telemedicine visits during the COVID-19 pandemic (8, 9). To our knowledge, studies comparing telemedicine to in-person care specifically for people living with lupus have not been conducted in the US and there is scarce knowledge regarding the benefits and limitations of telemedicine in patients with SLE especially in those from under-resourced backgrounds. To gain in-depth knowledge about the potential benefits, limitations, and acceptance of telemedicine in the management of SLE we conducted a qualitative study of patients with SLE receiving care at a safety net institution and their clinical team members.

## Methods

### Design

We conducted semi-structured interviews to identify acceptance, benefits, and limitations of telemedicine care for people with SLE. Methods and results are reported according to the Standards for Reporting Qualitative Research (SQPR) (10).

### Qualitative approach and research paradigm

We used a phenomenology approach to elicit the experiences and views of patients living with SLE from under-resourced communities and their clinical team members in regards to telemedicine. We chose a constructivist paradigm to understand how participants in telemedicine visits experienced their use of telemedicine, their attitudes and concerns, and the potential impact of telemedicine care on their disease management.

### Researcher characteristics and reflexivity

The research team consisted of nine researchers (SBr, SKA, KF, MM, SBo, MLO, MSA, MD, JY) including three rheumatology fellows (SBo, KF, MM) and six physicians (SBr, SKA, MLO, MSA, MD, JY). The team had prior experience with telemedicine and routinely cared for patients with rheumatic diseases. Patient interviews were conducted by SBr, KF, and MM, who only interviewed those patients for whom they had not participated directly in their care to prevent social desirability bias. The clinical team members were interviewed by KF and MM. Transcripts, coding, and thematic analysis were conducted by SBr, SBo, and SKA.

### Context

The study was conducted at a Harris Health rheumatology clinic in Harris County, Texas. Harris Health is a safety net inpatient and outpatient health system in which 54% of patients do not have insurance and rely on a county publicly issued insurance. In order to qualify for the publicly issued insurance, the patient needs to provide proof that the household income does not exceed 150% of the federal poverty level (11). A prior study conducted by our group found that between 2018 and 2020, there were 156 unique patients with SLE seen in the Harris Health Rheumatology clinic with 771 in-person visits and 182 telemedicine visits conducted. The clinic is staffed by 7 rheumatology attendings (12).

### Sampling strategy

We enrolled English and Spanish-speaking patients, rheumatologists, nurses, and medical assistants from the clinic to obtain a broad perspective on the topics discussed. At least two participants per group were interviewed. Enrollment was stopped once no new themes were identified (thematic saturation) (13). Interviews were conducted between December 2023 and March 2024. We selected participants by convenience and purposive (see below) sampling during routine clinic visits. All participants were ≥18 years of age.

**Patients**: Patients were identified through manual review of clinic charts by clinical team members and were required to be ≥18 years of age, have a diagnosis of SLE using the 2019 American College of Rheumatology or European League Against Rheumatism criteria, have low disease activity, defined as a SLE Disease Activity Index of less than 6, as this is the patient population that the research team thought might be most appropriate for telemedicine visits, and have prior experience with telemedicine with either a rheumatologist or a primary care physician (14, 15). During routine clinical visits, eligible participants were approached in person by a member of the research team after their clinical visit. No financial or material incentives were offered for participation in the study. We approached seven patients: six, including two Spanish-speaking patients agreed to participate. Thematic saturation with patients was reached after six interviews.

**Healthcare team**: We approached eight rheumatology care team members for interviews, all of whom agreed to participate (two nurses, two medical assistants, three rheumatologists, and one rheumatology fellow). All participants had previous experience with telemedicine. Rheumatology care team members were invited to participate during working hours through direct invitation by the research team.

### Data collection instruments

We conducted interviews in a semi-structured format and encouraged open discussion (Supplementary Material). We used the Levesque conceptual framework for healthcare access to develop our interview guide (16–18). This framework includes five domains related to services that influence health care access including approachability, acceptability, availability and accommodation, affordability, and appropriateness (18). Approachability refers to the ability of individuals with health needs to recognize that healthcare services exist and can be reached as well as have a positive impact on their health. Access to care can be improved with more approachable services by providing detailed information about treatments and services as well as engaging the community in outreach programs regarding health services. Acceptability refers to the social and cultural factors that influence whether people are willing to accept the aspects of a healthcare service. Acceptability can be applied to improve access to care by ensuring that healthcare services are culturally and socially aligned with values of the community, making people more willing to utilize these services. Availability and accommodation relates to health services being able to be reached both physically and in a timely manner. More available and accommodating health services such as telemedicine appointments can improve access to care for all patients including those with impaired mobility and limited access to transportation. Affordability refers to the financial capacity of people to spend resources and time to utilize appropriate healthcare services. Affordability can be applied to improve access to care by ensuring that health services are priced within the economic capacity of an individual, allowing them to allocate their resources and time to receive appropriate care. Appropriateness relates to the effectiveness and quality of health services. Opportunity to utilize high quality and effective services improves access to care. It additionally proposes five corresponding abilities of people: ability to perceive, ability to seek, ability to reach, ability to pay, and ability to engage. Barriers to access can arise from deficiencies in services or individuals' abilities (18). We also reviewed previous qualitative studies in telemedicine in populations other than SLE and developed a list of broad key concepts exploring potential benefits, pitfalls, and concerns towards telemedicine (19–22). Finally, we asked questions that specifically pertain to the care of SLE such as impact of telemedicine on flares and on adherence to laboratory studies. The questions for clinical team members and patients covered similar content (Supplementary Material). Baseline demographics including age (or years of practice), sex, race, ethnicity, and duration of lupus were collected.

### Data collection methods and technologies

Three researchers (SBr, KF, MM) conducted 1-on-1 interviews in a private room after patients had completed their regularly scheduled clinic visit. Clinicians were interviewed in patient rooms as well. All participants were instructed that interviews would be up to one hour in duration. The researchers were clinicians that are not involved in the patient's care and were trained by an expert in qualitative methodology (MLO). Interviews were anonymized. Digital audio recordings of the interview were obtained and transcribed verbatim and verified by one researcher (SBr) for accuracy. Spanish interviews were conducted by a bilingual researcher (SBr) and transcribed back to English for analysis.

### Data processing and units of study

Thematic saturation was reached after a total of 6 interviews with patients and 8 clinical team members. Thematic saturation was reached independently in each group. The units of analysis were the complete responses or phrases to interview questions. Transcripts were anonymized using identification codes and then transferred to the web application Dedoose to analyze the data (23).

### Data analysis

Thematic analysis was used. First, two researchers (SBr, SBo) familiarized themselves with the data: after three patient interviews the two researchers read transcripts to determine if codes were similar and created a preliminary code book. Additional sets of interviews were conducted until saturation was reached. Afterwards, two authors (SBr, SBo) individually performed initial coding of the data by assigning labels to phrases from transcripts. After coding, the researchers discussed direct quotes and formulated a coding scheme. We used a combination of inductive and deductive coding to identify subthemes and themes. We used the conceptual framework to identify broad themes, however, we also utilized a ground-up approach to identify new themes brought up by participants—specifically as it pertains to telemedicine and the care of patients with SLE. Afterwards, direct quotes that best described the themes and subthemes were selected. Coding was again reviewed to ensure that thematic saturation was reached.

### Techniques to enhance trustworthiness

To enhance the generalizability of the study findings, we interviewed a heterogeneous group of participants. To ensure trustworthiness, the data was coded by two separate researchers. Discrepancies were discussed and resolved through consensus with a third party (MLO) when consensus could not be reached by discussion. We also performed triangulation of data by examining the results from the interviews from clinical team members, patients, and the existing data from our literature search.

### Ethical issues pertaining to human subjects

The study was approved by the Baylor College of Medicine Institutional Review Board. Patients and clinical team members provided informed written consent.

## Results

### Participants

We conducted 14 interviews (six patients and eight clinical team members. The duration of interviews ranged from 6 min to 23 min. The characteristics of participants are shown in Table 1. The patients ages ranged from 24 to 67 years, all were female, and there were four White patients (66%), two Black patients (33%), and three Hispanic patients (50%). Clinical team members were predominantly female (*n* = 6, 75%), Asian (*n* = 6, 75%), with a wide range of years in practice (1–32 years).

**Table 1:** Participant characteristics.

Characteristics	Number
Patients	6
*Age (median, range)*	44 (24–67)
*Race (N, %)*	
White	4 (66)
Black	2 (33)
*Ethnicity (N, %)*	
Hispanic	3 (50)
*Sex, Female (N, %)*	6 (100)
*Disease Duration (median, range in years)*	9 (4–28)
*Interview Duration (range in minutes)*	6–23
**Clinical team member**	8
*Type (N, %)*	
Rheumatologists	3 (37.5)
Fellow	1 (12.5)
Medical Assistant	2 (25)
Registered Nurse	2 (25)
*Sex, Female (N, %)*	6 (75)
*Race (N, %)*	
Asian	6 (75)
Black	2 (25)
*Years in Practice (range)*	1–32
*Interview Duration (range in minutes)*	7–18

### Themes

**Figure 1:**
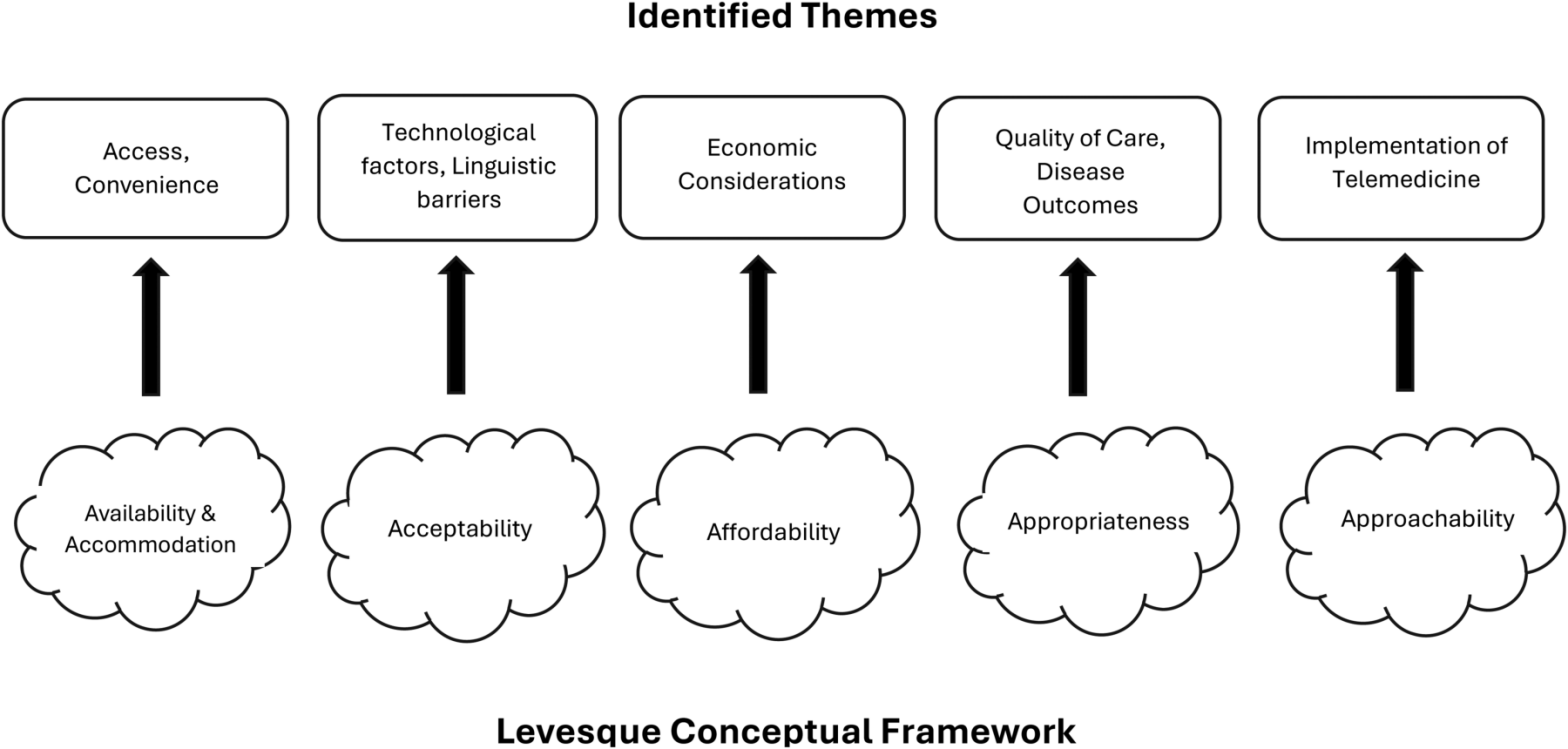
Relationship of identified themes to the levesque conceptual framework.

One hundred and fifty-one coded passages were identified. From these codes, five major themes were considered as major domains, including: (1) Access and Convenience (Availability and Accommodation), (2) Technological and Linguistic Barriers (Acceptability), (3) Economic Considerations (Affordability) (4) Quality of Care and Disease Outcomes (Appropriateness) (5) Implementation of Telemedicine (Approachability) (Figure 1). Themes were coded according to the Levesque conceptual framework (Table 2). Both clinical team members and patients identified similar issues.

**Table 2:** Relationship of levesque conceptual framework domains to themes identified and a description of the relationship.

Levesque	Themes identified	Description
Availability and Accommodation	Access	Telemedicine was perceived as more available and accommodating than in person visits because of improved geographic access and increased access to care in patients with mobility issues.
	Convenience	
Acceptability	Technological Barriers	Digital literacy especially in older patients as well as language discordance between provider and patient were identified as barriers that might affect acceptability of telemedicine to patients and clinical team members.
	Linguistic Barriers	
Affordability	Economic Considerations	Telemedicine was generally viewed as a more affordable option compared to in person visits due to reduced visit fees as well as costs associated with driving and parking.
Appropriateness	Quality of Care	Telemedicine was generally viewed as most appropriate for stable patients and not for patients having a disease flare or new patients. There were concerns regarding negative impact on rapport and lack of a physical examination.
	Disease Outcomes	
Approachability	Implementation of Telemedicine	Patients and clinical team members identified providing patients with adequate training on telemedicine platforms and establishing protocols and support for addressing technical issues that arise during virtual visits as factors that could make telemedicine more approachable.

## Theme 1: access and convenience (availability and accommodation)

According to Levesque's conceptual framework, availability and accommodation relates to health services being able to be reached both physically and in a timely manner. More available and accommodating health services such as telemedicine appointments can improve access to care for all patients including those with impaired mobility and limited access to transportation (17). Four out of six patients, and six out of eight clinical team members felt that telemedicine would increase access to care and would be more convenient for patients. Subthemes identified included specific logistic barriers to in-person routine care that could be resolved with telemedicine such as geographic access (e.g., transportation issues related to distance from patients' residence to the clinic, having to find a ride, or having to take public transportation), other miscellaneous issues include bad weather, childcare, and other work/school responsibilities.

Telemedicine is good because of issues with transportation, I don't have to have a car or find a ride. (Patient 5)

My transportation is provided by my insurance company, and we're only allowed so many rides per year … I wouldn't have to use that ride. (Patient 1)

Video visits would benefit patients that need someone else to drive them, or if they live far away, or if they don’t have a car, can't afford the bus, etcetera. (Rheumatologist 3)

…if patients don't have to travel as far to access rheumatology, you can imagine access is hard if they're far away without a lot of rheumatologists that take noninsured patients. (Rheumatologist 3)

The second subtheme identified was mobility and physical access. Some patients interviewed had physical impairments, and others were worried about themselves or family members being at high risk of infection, which limited them from coming to in-person clinic visits. Clinical care members also identified telemedicine as being potentially beneficial to patients with physical impairments.

My 2-year-old son is on chemotherapy … I would rather just stay at home and avoid people. (Patient 3)

I have this one patient who literally had a compression fracture of his back. He wasn't able to come in, so I was able to call him. (Fellow)

Sometimes we get patients that are really, really ill and aren't able to come in. (Medical assistant 2)

Video visits come in handy because I am disabled and I have an oxygen tank. (Patient 4)

If someone is disabled and needs an ambulance to come, they won't have to call and schedule that. (Rheumatologist 3)

The third subtheme included other miscellaneous logistic barriers to in-person visits, such as bad weather, childcare, and work/school responsibilities.

Video visits are much easier on the days I can't come in or the weather is really bad for me. (Patient 4)

There have been visits I didn't make because of the weather. (Patient 2)

It is way easier on me to do the visits at home for my kids. (Patient 3)

Things like childcare or your job can limit your ability to come to clinic (Rheumatologist 2)

## Theme 2: technological and linguistic barriers (acceptability)

According to Levesque's conceptual framework, acceptability refers to the social and cultural factors that influence whether people are willing to accept the aspects of a healthcare service. Acceptability can be applied to improve access to care by ensuring that healthcare services are culturally and socially aligned with values of the community, making people more willing to utilize these services (17). We identified technological and linguistic barriers as affecting the acceptability of telemedicine. Technological factors were mentioned by four out of six patients, and by all clinical team members. Subthemes include patient-related technical issues and telemedicine infrastructure. Digital literacy was a concern for both patients and clinical team members. Clinical team members were particularly concerned that older patients would have issues with telemedicine. In this under-resourced setting, there was concern that patients may not have adequate technology to be able to participate in telemedicine.

I have difficulties using the phone sometimes. I couldn't enter the application once. (Patient 6)

I think it's possible there is a barrier in that the patient needs a phone and a camera and knows how to use it. (Rheumatologist 3)

I think with the older generation they aren't tech friendly, it's going to be difficult for them. (Nurse 2)

Most people who do telemed visits will do it over the phone and that can sometimes not be great because it requires an app and not just a website. (Rheumatologist 2)

With our population of patients, they may not have access to cellphones that are smart phones or tablets. (Medical assistant 2)

The second subtheme identified is telemedicine infrastructure which includes equipment not working as expected, inadequate environment to conduct telemedicine visits (i.e., lack of privacy), and language barrier between patients and clinical team members. Internet connectivity, microphone or camera not working, and phones or other electronic devices running out of battery power were all concerns that were brought up by both patients and clinical team members.

The only issue is some parts of my house have a bad internet connection. (Patient 5)

I did have a call drop because the patient's phone ran out of battery power. (Fellow)

Another barrier is if you're unable to hear the patient or connection issues. (Nurse 1)

Clinical team members and patients were concerned about the suitability of the environment to conduct telemedicine including patients taking calls in loud environments, with other people in the background, while driving, or being in a location other than at home during the visit.

The other problem is I have a lot of dogs and they will bark and cause a lot of noise. (Patient 5)

A lot of times people are doing telemedicine visits while driving, so that's another danger … They need to be in a private location. (Rheumatologist 3)

…sometimes patients would do visits in their car and we couldn't do everything or sometimes patients would be with a number of other people and didn’t want to do things either. (Rheumatologist 2)

Four out of eight clinical team members were concerned about language barriers and incorporating an interpreter into the telemedicine visit. Clinical team members were concerned that the non-English speaking patients' experience may not be adequately represented by a virtual interpreter. However, these concerns were not conveyed by the Spanish-speaking patients.

Sometimes you have to wait for an interpretation. You don't get it immediately. Plus some languages you don't get. (Medical assistant 1)

Even through a translator, I don’t think it will be 100% clear for the provider. Yeah, even through the interpretation group. I don't think the real experience will be represented by the translator. (Medical assistant 1)

Language barrier is huge because getting a translator online and integrating it into the visit is difficult. (Rheumatologist 1)

## Theme 3: economic considerations (affordability)

According to Levesque's conceptual framework, affordability refers to the financial capacity of people to spend resources and time to utilize appropriate healthcare services. Affordability can be applied to improve access to care by ensuring that health services are priced within the economic capacity of an individual, allowing them to allocate their resources and time to receive appropriate care (17). Telemedicine overall was viewed as less costly to patients compared to in-person visits. It was felt that patients could save money on transportation, gas, and parking fees.

The visits may be cheaper too, because telemedicine charges less. (Medical assistant 1)

The cost might be a little bit more affordable if it's telemedicine vs. in-person. (Nurse 1)

Telemedicine would make it easier for me to save gas going back and forth to clinic visits. (Patient 3)

The gas prices are up right now and they go up and down. These small things matter quite a bit to access to care. Not only the gas, they also have to drive, they have to pay parking fees (Rheumatologist 1)

On the other hand, a minority of physicians were also interested in knowing more about physician reimbursement and how it would be affected for telemedicine visits compared to in-person visits.

…also has to do with reimbursement because reimbursement for telemedicine is less than in-person. (Rheumatologist 2)

## Theme 4: quality of care and disease outcomes (appropriateness)

According to Levesque's conceptual framework, appropriateness relates to the effectiveness and quality of health services. Opportunity to utilize high quality and effective services improves access to care (17). Perceptions about quality of care provided through telemedicine was mainly a concern discussed by clinical team members. Two subthemes identified were the patient-physician relationship and the medical evaluation (e.g., physical exams, labs, and accuracy of diagnosis and management). Three out of eight clinical team members felt that telemedicine could harm the clinician-patient relationship. They cited challenges such as establishing rapport, obtaining patient history, and limited non-verbal communication.

Patients get a good rapport from our doctors just being in-person, getting to know each other. (Medical assistant 2)

Seeing somebody face to face, looking at their emotions and also their expressions and their tone of voice really helps with getting the full picture. (Fellow)

The second subtheme was the medical evaluation including the physical exam, laboratory tests, accuracy of diagnosis, and appropriateness of management. Concerns regarding lack of physical exam were mentioned by seven out of eight clinical team members, and three out of six patients.

Sometimes the pain is really bad or I’m having a flare and I want a doctor to examine me. (Patient 5)

You can't feel the joints, you can't watch them walk very easily. So there are limitations on the physical exam. I’d say maybe you can alternate physical in-person and on telehealth. (Rheumatologist 3)

Maybe as a provider we get too comfortable with just taking whatever the patient is telling us for you know, face value rather than actually feeling the joints and examining the skin. (Fellow)

There is a risk that something is missed because we didn't do a physical exam. (Rheumatologist 2)

Another common concern was patients' continuing to get their laboratory testing performed as many patients currently get them drawn on the same day as their appointment. Two patients did not see the benefit of telemedicine if they still had to come in-person to get their labs drawn, whereas two others did not see a problem with coming in for laboratory testing on days other than the clinic visit.

I don't see the benefits in being able to talk to my doctor over video if I still have to come here to get blood work. (Patient 1)

I don't think it would actually affect me getting my labs done. (Patient 6)

I'm OK with you doing telemed as long as you're doing all the things for a telehealth visit to be helpful to you and part of that is getting your labs done at appropriate intervals. (Rheumatologist 2)

If patients are not reliable on getting their labs, I often switch them to an in-person visit to get labs, because if they're here they are more likely to get the labs and urine testing. (Rheumatologist 3)

Patients and clinical team members generally viewed telemedicine as the most appropriate for stable follow up patients. There were mixed opinions regarding whether telemedicine should be used for patients experiencing flares. Overall, only two out of six patients, and two out of eight clinical team members felt telemedicine could be used in patients with SLE flares, as patients could be seen more quickly and more comfortably. Most preferred in-person visits for patients with flares, citing the importance of a physical examination in this setting.

If you're going through a flare and you need to see your doctor, sometimes you can't always wait a week or two. (Patient 4)

…if I'm really in pain, which I've only missed one rheumatology visit, and so, for example, that day I couldn't come in because I was feeling horrible, if I would have been offered a video visit I still could have seen my doctor. (Patient 2)

I think telemedicine visits should be used when certain instances occur like if they have a flare up, but I don’t think it should be used for regular visits. (Nurse 1)

I think it's convenient because I know for people with lupus, especially when they have flare ups and they’re miserable they would rather talk to the doctor on video than come in. (Nurse 2)

Both patients and clinical team members felt telemedicine could be an option for patients with low disease activity. Clinical team members did not think telemedicine should be used for new patient visits.

Video visits should only be done when patients feel well. When we are stable and without symptoms. (Patient 5)

…having well established patients who have minimal disease activity, incorporating telemedicine into their ongoing care. (Rheumatologist 2)

…it might actually be helpful in patients who have very stable disease activity… there are limitations, but I think overall very helpful in a chronic disease process requiring frequent visits like lupus. (Rheumatologist 3)

All rheumatologists believed that telemedicine could improve patient adherence with visits and drug therapy. They also felt that telemedicine visits could be structured to better assess comorbidities that are associated with SLE. Medication adherence in the telemedicine setting vs. the in-person setting was not viewed as a barrier given patients within Harris Health have the option to have all of their prescriptions mailed directly to their home or if they prefer, they can pick up their prescriptions from one of the sixteen Harris Health outpatient pharmacies located across Harris County, Texas.

…would be a nice way to increase compliance and reinforce other things that come along with lupus, you know, be it depression or concern for risk of stroke and other comorbidities, which we often don't address enough in our visits. (Rheumatologist 2)

…It could increase their compliance to meds and their compliance to following up. (Rheumatologist 3)

## Theme 5: implementation of telemedicine (approachability)

According to Levesque's conceptual framework, approachability refers to the ability of individuals with health needs to recognize that healthcare services exist and can be reached as well as have a positive impact on their health. Access to care can be improved with more approachable services by providing detailed information about treatments and services as well as engaging the community in outreach programs regarding health services (17). Patients and clinical team members were asked about what could be helpful in implementing a telemedicine program. Most patients felt that being taught how to use the application in advance could be helpful.

…you would have to conduct a class on how us as clients answer the phone and what we need to do. (Patient 1)

It would be nice if someone could explain it first. (Patient 6)

Clinical team members also felt that teaching the patients how to use the application in advance, either through a tutorial or by in-person teaching at the end of their current in-person visit, would be beneficial in implementing telemedicine and make it more approachable to patients Ensuring the patient has a working phone number or email address, and that their voice mail is set up prior to the telemedicine appointment were viewed as essential. Having a protocol for unexpected technological mishaps was also cited as important.

We should make sure there is a telephone number that works, make sure that it is in service and make sure that their voice-mail works. (Fellow)

Sometimes there's also technological difficulty, so unless we had a way of establishing a protocol for XY and Z is happening with technology, especially on the patient end, it would be a barrier. (Rheumatologist 2)

I think we should educate them during their current clinic visit. (Rheumatologist 1)

The only thing I'd say is definitely some communication with nursing staff to the patients as well, because I know there's also some individuals who are not very tech savy and need assistance with that. (Nurse 1)

## Discussion

To our knowledge, this is the first qualitative study using in-depth interviews to examine perceptions about telemedicine in a US clinical setting providing care to under-resourced patients with SLE. We triangulated the results by collecting and analyzing information from both English-speaking and Spanish-speaking patients, and members of the healthcare team performing different roles (rheumatologists, nurses, medical assistants, and a rheumatology fellow). Our objective was to gain an open and broad perspective on the advantages and challenges of using telemedicine to provide care for under-resourced patients with SLE. The five key themes identified and their corresponding domains from Levesque's conceptual framework for healthcare access included (1) Access and Convenience (Availability and Accommodation), (2) Technological and Linguistic Barriers (Acceptability), (3) Economic Considerations (Affordability), (4) Quality of Care and Disease Outcomes (Appropriateness), and (5) Implementation of Telemedicine (Approachability). Our analysis showed that telemedicine could improve access to care and adherence to clinic visits by reducing the barriers associated with socioeconomic factors. On the other hand, barriers to telemedicine identified included digital literacy, concern about negative impact on physician-patient relationship, and language discordance.

Levesque's conceptual framework proved to be highly effective for our qualitative telemedicine study of patients with SLE and their clinical team members. By categorizing our identified themes into specific domains, we were able to systematically analyze various aspects of telemedicine. For instance, the framework's focus on availability and accommodation helped us understand the importance of access and convenience for patients. Similarly, the acceptability domain allowed us to highlight technological and linguistic barriers that patients and their clinical team members faced when using telemedicine. The framework also facilitated a comprehensive examination of economic considerations under the affordability domain, and it provided a structured way to assess quality of care and disease outcomes through the appropriateness domain. Lastly, the approachability domain was instrumental in identifying optimal strategies for future telemedicine implementation. Overall, the Levesque framework enabled a thorough and organized exploration of the multifaceted impacts of telemedicine on patients with SLE and their care teams.

Patients and clinical team members believed that telemedicine can improve access to care and enhance convenience for patients, particularly for those with transportation difficulties, mobility issues, and geographic barriers. Moreover, telemedicine was generally viewed as a strategy to decrease total out of pocket expenses for patients including gas expenses, parking fees, and office visit fees. Childcare needs, work/ school responsibilities, and caring for ill family members were other factors highlighted as influencing patients' ability to access care, further underscoring the role for telemedicine in this patient population, which is relatively young, during productive years of their lifespan. Telemedicine has been shown to reduce overall out of pocket expenses, travel time, and time missed from work or school in other patient populations (24).

While telemedicine was viewed as having many potential benefits, there were concerns about digital literacy and access to technology. Older patients were perceived to face challenges in adapting to telemedicine, raising concern about equitable access amongst all age groups. A cross-sectional survey of rheumatology patients found a negative correlation between increasing age and access to technology including front facing camera, telephone, and stable internet connection (19). Older patients were also less likely to believe that their needs could be met through telemedicine (19). Other barriers identified included issues with telemedicine infrastructure including poor internet connection, loud and non-private environments, and equipment not working adequately. Patient privacy and safety as it pertains to telemedicine remains a concern. A recent systematic review identified three risk factors associated with privacy and security in telehealth practice including environmental factors (e.g., lack of private space for vulnerable populations), technology factors (e.g., data security issues and limited access to the internet), and operational factors (e.g., technology accessibility, training, and education) (25). Language barriers between clinical team members and patients was perceived as a significant barrier for clinical team members, but interestingly, the Spanish-speaking patients who were interviewed did not see language as barrier to telemedicine care. A prior telemedicine study did find that fewer non-English speaking patients were seen via telehealth compared to in-person visits, which possibly reflects variable patient and/or provider comfort with virtual visits depending on language fluency, and challenges with the availability and addition of phone interpreters to virtual visits (20).

The perceived impact of telemedicine on the quality of care in patients with SLE varied amongst participants. A minority of clinical team members expressed concern about its negative impact on the physician-patient relationship, and the limited ability to conduct physical examinations. Both patients and clinical team members also expressed concern about patients continuing to get their laboratory testing performed at appropriate intervals if they received telemedicine care. Other patients and clinical team members did not perceive telemedicine to have a negative impact on the patient-physician relationship or in their ability to have labs done at the appropriate intervals. Studies in patients with rheumatic diseases have conflicting findings on this topic, with one study raising concerns, while another did not find a negative impact on the medical relationship (19, 26).

A mixed methods study using surveys and in-depth patient interviews in a U.K. population with various rheumatic diseases found that patients and physicians overall rated telemedicine worse than face-to-face in almost all categories with building trusting medical relationships and assessment accuracy as major concerns (26). However, they did find telemedicine to be equally or more acceptable in stable patients who are secure in their relationship with their rheumatologists (26). Notably, socioeconomic status and associated constraints like transportation or child-care needs were not explored in that study. In our study, patients and clinical team members generally perceived telemedicine to be appropriate for stable patients with low disease activity. However, the suitability of telemedicine for SLE flares varied amongst both the clinical care team members (physicians vs. ancillary staff) and patients. Physicians generally felt disease flares were better managed through in-person visits, whereas some patients perceived telemedicine to be beneficial for flares because of quicker access to a physician. Clinical care team members generally agreed that telemedicine would not be appropriate for new patient visits. Consistent with past literature, individual choice and careful patient selection for telemedicine are essential to ensure patient safety and acceptability (22, 26). While telemedicine appears to be best suited for patients with stable disease, future research should examine whether certain components of telemedicine, like patient education or remote monitoring, could be beneficial for patients with active disease who may feel too unwell to attend their clinic visit.

Several patients and clinical care team members made recommendations to facilitate the successful implementation of telemedicine in under-resourced patients with SLE. These included providing patients with adequate training on telemedicine platforms either through tutorials or in-person training after a clinic visit, ensuring access to adequate technology, and establishing protocols and support for addressing technical issues that arise during virtual visits. Compared to the general population, under-resourced groups encounter unique challenges including limited digital access, lower health and digital literacy, language barriers, and environmental constraints. Addressing these disparities requires equity-focused approaches such as culturally and linguistically tailored communication, digital navigation support, flexible care delivery models, and community-engaged outreach strategies.

To our knowledge, this is the first US study to examine perceptions about telemedicine in under-resourced patients with SLE and their healthcare team members. Strengths include purposive sampling to include a broad range of perspectives from patients including Spanish-speaking patients and from their clinical care team fulfilling different healthcare roles, therefore contributing to the triangulation of results. Moreover, our participants were highly diverse with respect to ethnicity, race, language-preference and age. Patients varied in the duration of disease, and clinical care members in their years in practice. We conducted face-to-face interviews, which helped build rapport and allow the interviewer to pick up on non-verbal cues, enriching the interview process. Our study had some limitations related to the generalizability of results. It was conducted at a single clinical setting. All patients were female, as are the majority of people living with SLE, so male patients' perspectives were not represented in our study. Notably, while SLE is less common among men, men who have SLE often have more severe disease compared to women (27). This difference in disease severity could lead to distinct healthcare needs, such as more frequent or intensive medical interventions, which may not be adequately addressed through telemedicine. Any extrapolation of this study's findings to male patients should be done with caution until further studies are done that assess the specific needs and experiences of men with SLE. There was less racial diversity in the clinical care members interviewed which may have impacted their perspectives regarding telemedicine, but the workforce in our clinic has a large majority of individuals from the groups interviewed, especially those from Asian ancestry. Although the sample size was small, we reached thematic saturation, and it was felt that additional interviews would not identify new themes. However, thematic saturation is a qualitative judgment and could vary based on the researcher's interpretation. We attempted to limit social desirability bias by not interviewing patients that were known to the research team, however, as patients were receiving care within the same department, there may still be a risk of bias. Participants who agreed to participate in interviews may have had more positive or negative experiences with telemedicine than those who declined to participate, which could have introduced selection bias. Finally, patients interviewed had low disease activity as we felt this was the most appropriate group to engage in telemedicine care, given safety concerns in patients with active disease. Therefore, the results cannot be generalized to patients with SLE with active disease since this population was excluded from the study. To enhance the generalizability of our findings and validate them across different populations, future research should aim to recruit patients outside of safety-net hospital settings, men, and continued enrollment of individuals from racially and ethnically diverse backgrounds and geographic locations.

Our study highlights the complex interplay of factors affecting the adoption of telemedicine in an under-resourced population with SLE. Telemedicine has the potential to improve access to care and improve adherence to clinic visits by reducing the burden of socioeconomic and logistic barriers. The insights gained from this study can aid in the development of structured telemedicine programs for under-resourced patients with SLE. Crucial implementation strategies include adequate education and technology training for patients and clinical team members, prior evaluation of patient access to the needed technology for telemedicine, availability of trained interpreters, protocols in place to resolve unexpected technical issues, planning for laboratory testing prior to the visit as needed, and patient preferences for telemedicine visits. In addition, more research is needed to determine which patient populations can be safely managed with telemedicine without a detriment to their clinical status. These findings will ultimately inform the development of a structured telemedicine program to improve adherence to clinic visits for patients living with SLE without compromising quality of care.

## Data Availability

The original contributions presented in the study are included in the article/Supplementary Material, further inquiries can be directed to the corresponding author.
