# Supplementary material for: Telemedicine for under-resourced patients with systemic lupus erythematosus: a qualitative study exploring the views and experiences of patients and their healthcare team
Source: Front Health Serv. 2025 May 22;5:1503881. doi: 10.3389/frhs.2025.1503881 (PMC12137260; doi:10.3389/frhs.2025.1503881)
Supplement: Supplementary file 1 [file Datasheet1.pdf]

Supplementary Material 1.

Semi-structured interview for patient participants.

Thank you for participating in this interview. We are in the process of implementing a video telemedicine program in patients living with lupus. Our goal is that in patients that have well-controlled disease and no medication changes within 6 months, we would offer every other visit to be a video visit. However, we would like to get our patients' perspectives and attitudes towards telemedicine prior to implementing this program.

Theme 1. Prior experiences with telemedicine

1) *What has been your experience of telehealth?*
2) *How often do you have video telemedicine visits?*
3) *Have you had any positive experiences with telemedicine?*
4) *Have you had any negative experiences with telemedicine?*
5) *Do you see telemedicine being a part of your care in the future?*

Theme 2. Perceptions about how telemedicine may impact their care, specifically as it pertains to lupus

6) *How do you feel like telemedicine has affected your care?*
7) *How do you feel that telemedicine has impacted your living with lupus?*
8) *Has telemedicine impacted how you get your blood work and urine studies done or medications?*
9) *Would telemedicine make it easier to attend clinic visits?*

Theme 3. Perceptions about how telemedicine may compare to usual care

10) *How do you feel that telemedicine has impacted your health care outside of lupus (such as primary care, visits with the OB)?*
11) *Has telemedicine impacted your ability to get vaccinations, screening tests (such as pap smears, mammograms, or colonoscopies), or regular blood work for diabetes and cholesterol?*

Theme 4. Potential benefits of telemedicine

12) *What are some potential benefits that you may see in telemedicine visits?*

13) *Does telemedicine help you to make your clinic appointments?*

14) *Are there any issues that prevent you from attending an in-person clinic visit (such as childcare, parking costs) that might be solved by having a telemedicine program?*

Theme 5. Potential concerns or harms with telemedicine

15) *Are there any harms you see with telemedicine?*

16) *How do you feel telemedicine might impact your ability to get blood draws and urine studies?*

17) *Do you feel like telemedicine will impact your care without your doctor being able to examine you in person?*

Theme 6. Preferences for participating in telemedicine visits

18) *Do you think that telemedicine should remain an option for patients with lupus?*

19) *How would you feel about implementing a telemedicine program for patients with lupus where if the disease is well-controlled, every other visit would be a video visit?*

20) *How do you think this might affect care?*

21) *Would you participate in a telemedicine lupus program? Why or why not?*

Theme 7. Barriers for participating in telemedicine visits

22) *What are some barriers you would see towards implementing a telemedicine program in patients with lupus?*

23) *Is there anything that could be done to make it easier to implement such a program?*

24) *Do you have any other thoughts or concerns?*

Supplementary Material 2.

Semi-structured interview for providers

Thank you for participating in this interview. We are in the process of implementing a video telemedicine program in patients living with lupus. Our goal is that in patients that have well-controlled disease and no medication changes within 6 months, we would offer every other visit to be a video visit. However, we would like to get our provers' perspectives and attitudes towards telemedicine prior to implementing this program.

Theme 1. Prior experiences with telemedicine

1) *What has been your experience of telehealth?*
2) *How often do you have video telemedicine visits?*
3) *Have you had any positive experiences with telemedicine?*
4) *Have you had any negative experiences with telemedicine?*
5) *Do you see telemedicine being a part of patients' care in the future?*

Theme 2. Perceptions about how telemedicine may impact their care, specifically as it pertains to lupus

6) *How do you feel like telemedicine has affected lupus patients' care?*
7) *Overall, do you think the implementation of telemedicine has been positive or negative for patients with lupus?*

Theme 4. Potential benefits of telemedicine

8) *What are some potential benefits that you may see in telemedicine visits?*
9) *In your experience, has telemedicine solved barriers for some patients that are unable to attend in-person visits (such as work, childcare)? Can you provide specific examples?*

Theme 5. Potential concerns or harms with telemedicine

10) *Are there any harms you see with telemedicine?*
11) *Do you think that patients not seeing a doctor in-person might affect their care?*

Theme 6. Preferences for participating in telemedicine visits

12) *Do you think that telemedicine should remain an option for patients with lupus?*
13) *How would you feel about implementing a telemedicine program for patients with lupus where if the disease is well-controlled, every other visit would be a video visit?*
14) *How do you think this might affect care?*
15) *Would you participate in a telemedicine lupus program? Why or why not?*

Theme 7. Barriers for participating in telemedicine visits

16) *What are some barriers you would see towards implementing a telemedicine program in patients with lupus?*
17) *Do you see digital literacy (patients ability to use phones, computers) or language as potential barriers for telemedicine?*
18) *Is there anything that could be done to make it easier to implement such a program?*
19) *Do you have any other thoughts or concerns?*
